# Differences in nature of electrical conductions among Bi_4_Ti_3_O_12_-based ferroelectric polycrystalline ceramics

**DOI:** 10.1038/s41598-017-03266-y

**Published:** 2017-06-23

**Authors:** Changbai Long, Qi Chang, Huiqing Fan

**Affiliations:** 1 Science and Technology on Plasma Dynamics Lab, Air Force Engineering University, Xi’an, 710038 P.R. China; 2 State Key Laboratory of Solidification Processing, School of Materials Science and Engineering, Northwestern Polytechnical University, Xi’an, 710072 P.R. China

## Abstract

Bismuth titanate Bi_4_Ti_3_O_12_ (BiT), was one of the most promising lead-free high-temperature piezoelectric materials, due to high Curie temperature (675 °C) and large spontaneous polarization (50 *µ*C/cm^2^); however, extensive studies had revealed that high leakage conductivity interferes with the poling process, hindering its practical applications. In this paper, an electrically insulating property was achieved by a low level Nb donor substitution to suppress a high level of holes associated with high oxygen vacancy concentration. Bi_4_Ti_2.97_Nb_0.03_O_12_ ceramic showed significant enhancements of electrical resistivity by more than three order of magnitude and activity energy with value >1.2 eV, which are significant for piezoelectric applications of BiT-based materials. However, pure and A_2_O_3_-excess (A = Bi, La and Nd; 3 *at* %) BiT ceramics, were mixed hole and oxygen ion conductors. Schottky barriers were both formed at grain boundary region and the sample-electrode interface, because of the existence of semiconducting bulk. Interestingly, the electron conduction could be suppressed in N_2_, as a consequence, they became oxide ion conductors with conductivity of about 4 × 10^−4^ S cm^−1^ at 600 °C.

## Introduction

Bismuth titanate Bi_4_Ti_3_O_12_ (BiT), has been widely studied as one of most important bismuth-layer-structured ferroelectrics (BLSFs), which consists of three-layer pseudo-perovskite (Bi_2_Ti_2_O_10_)^2−^ units sandwiched between (Bi_2_O_2_)^2+^ layers along the *c* axis^1–5^. BiT shows a high Curie point (*T*_c_) of 675 °C and a large spontaneous polarization (*P*_s_) of about 50 µC/cm^2^ ^6, 7^. It is well known that Bi_2_O_3_ vaporization with generations of V_Bi_′′′ (bismuth vacancy) and V_O_^••^ (oxygen vacancy) is a severe problem in the preparation of BiT-based materials, which largely affect electrical properties of them. High leakage conductivity closely related to the existence of V_O_^•• ^^8–10^, would interfere with the poling process by an applied field. This, combined with a pinning effect on domain walls by point defects or defect dipoles [*e*.*g*., (V_Bi_′′′-V_O_^••^)′]^11–15^, results in a low remnant polarization (*P*_r_ < 8 μC/cm^2^) for BiT thin films and ceramics^16–20^. Furthermore, a low poling field (<10 kV/cm) leads to a low piezoelectric activity (*d*_33_ ≤ 8 pC/N), which has been an obstacle for piezoelectric applications of BiT-based materials^4–6^. Great efforts have been made to solve such problems in BiT materials as mentioned above. Isovalent substitution for Bi^3+^ by lanthanoid cations (La^3+^, Pr^3+^, Nd^3+^, *etc*.) at the perovskite A site has been found to be an effective way to reduce V_O_^••^ and V_Bi_′′′ concentrations in the perovskite layers^3, 21–23^. On the other hand, B-site donor substitution such as W^6+^, Nb^5+^ or Ta^5+^, can significantly decrease the bulk conductivity and enhance ferroelectric and piezoelectric properties of the samples^11, 12, 24–27^. Also, excess raw oxides (*e*.*g*., Bi_2_O_3_ and CeO_2_) have been recommended by compensating the Bi_2_O_3_ vaporization^28–30^.

It is distinct that a problem that must be addressed in the development of BiT-based sensors is the origin of high leakage conductivity of materials. Takahashi *et al*. revealed that BiT single crystals were oxide ionic and *p*-type mixed conductors^31^. They suggested that V_O_^••^ and V_Bi_′′′ preferentially existed in the perovskite layers, which could be effectively suppressed by the A-site La or the B-site donor substitution; thus the decreases in electronic and ionic conductivities were achieved, along the *a*(*b*) axis in BiT single crystals^31–33^. But for ceramics, the grain boundary is likely to act as a source or sink for defects (oxygen vacancies)^34–38^. Consequently the ceramics other than single crystals, are often electrically heterogeneous. BiT ceramics also showed oxide ionic and *p*-type mixed conduction, where the electrical properties of the boundaries were pronounced^39, 40^.

Impedance spectroscopy has been widely used to investigate AC conduction behaviors of crystalline, polycrystalline and amorphous materials^41^. Commonly, electrode, grain boundary and grain components together contribute to the conductions of polycrystalline materials^41–43^. In this paper, impedance data of BiT-based ceramics including BiT, BiT-A (3 *at*.% excess A_2_O_3_; A = Bi, La and Nd), BLT (Bi_3.25_La_0.75_Ti_3_O_12_) and BiT-Nb (Bi_4_Ti_2.97_Nb_0.03_O_12_), were studied systematically, to investigate the effects of different modifications on heterogeneous structure and electrical conductivity. Also, electrical conduction behaviors of them as functions of temperature and atmosphere were elaborated.

## Results

### Structure and compositional analysis

**Figure 1:**
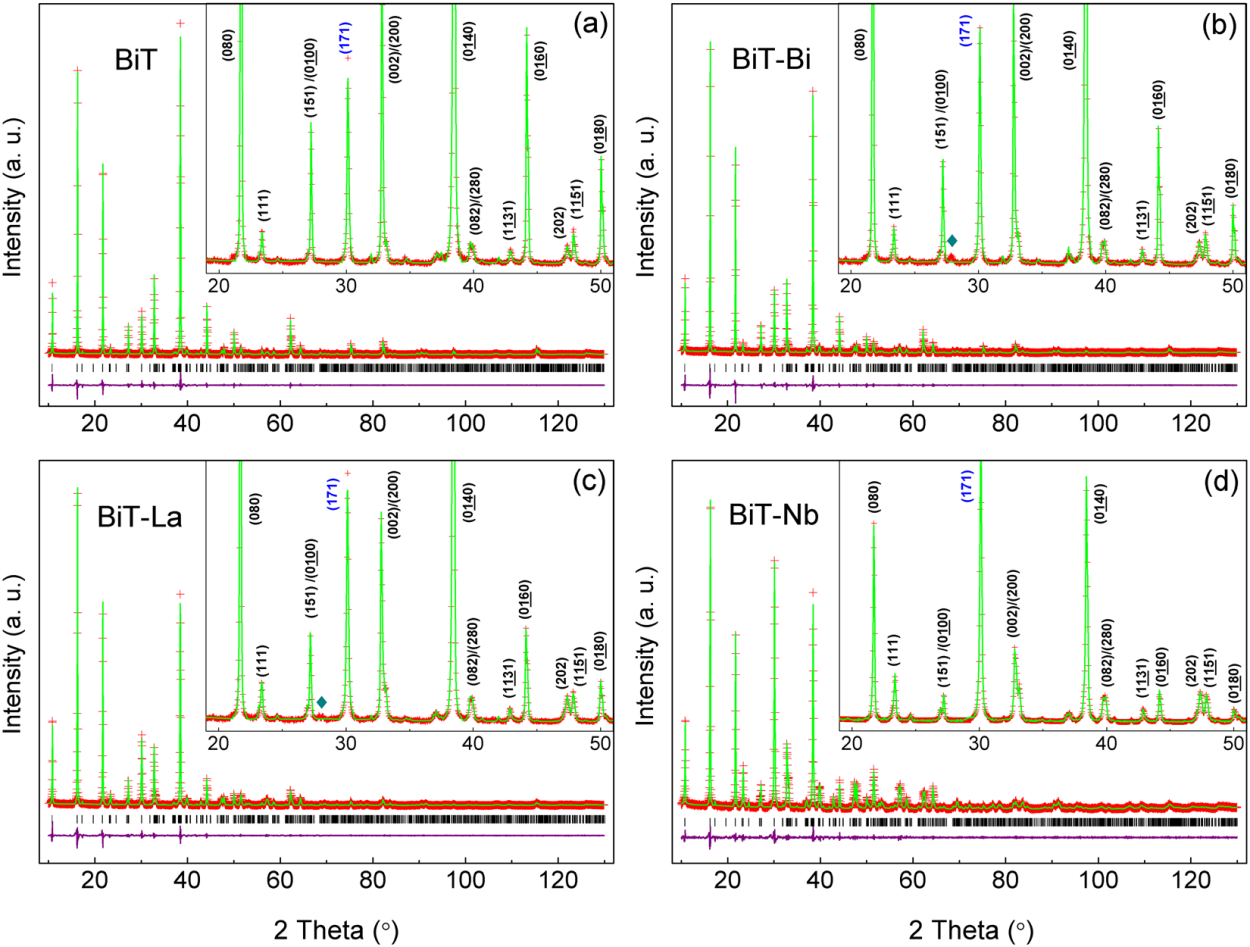
Powder diffraction refinements for (**a**) BiT, (**b**) BiT-Bi, (**c**) BiT-La, and (**d**) BiT-Nb ceramics. In (**b**) and (**c**), “♦” denoted as an impurity phase, Bi_2_O_3_.

Powder diffraction refinement with a Le-Bail fit (*GSAS-EXPGUI* program)^44, 45^ was carried out to characterize crystal structures of the prepared oxides, as shown in Fig. 1. The orthorhombic *Aba*2 (ICSD #240210) transformed from *B*2*cb* (*Aba*2: *abc* = *B*2*cb*: *b*′*c*′*a*′) was used as an initial structural model. For each composition, the calculated data well agrees with the experimental ones, and the reliability factors (*R*_wp_, *R*_p_ and reduced χ^2^; Table 1) are reasonable. However, the refined results reveal small amounts of Bi_2_O_3_ in BiT-A (*e*.*g*., BiT-Bi and BiT-La), corresponding to the small diffraction peak at 2*θ* = ∼28° (insets of Fig. 1b and c). In addition, the (0*l*0) diffraction peaks in BiT, BiT-Bi and BiT-La, *e*.*g*., (060), (080) and (0140), are abnormally intensive, while the major peak (171) is severely supressed (Fig. 1a–c). Absolutely, they were highly textured in the *c* axis direction. However, the texture is not pronounced in BiT-Nb (Fig. 1d). The degree of texture can be expressed by using Lotgering orientation factor (LOF), *f* ^46^. For (0*l*0) preferred orientation, *f* is defined as following equations:

1 $$f=\frac{P-{P}_{0}}{1-{P}_{0}}$$

where $P=\sum {I}_{(0l0)}/\sum {I}_{(hkl)}$(target sample) and $P=\sum {I}_{(0l0)}/\sum {I}_{(hkl)}$ (standard random sample, PDF#35–0795). For BiT and BiT-A, highly *c*-oriented structure results in high *f* value, being around 0.9, much higher than that for BiT-Nb (0.54). Table 1 lists refined lattice parameters of BiT, BiT-A and BiT-Nb. BiT-Nb shows larger lattice parameters (*a*, *b* and *c*) and unit cell volume (*V*) than BiT, which should be the results of incorporation of Nb^5+^ with larger size at B site (Nb^5+^: N0.64 Å, Ti^4+^: 0.605 Å; 6 CN)^47^. BiT-Bi and BiT-La show increased *V*, however, this value for BiT-Nd decreases.

**Table 1:** Crystal data, lattice parameters (*a*, *b*, *c*) and unit cell volume (*V*) of BiT, BiT-A and BiT-Nb, whose orthorhombicity is defined as 2(*c* − *a*)/(*c* + *a*).

Composition	BiT	BiT-Bi	BiT-La	BiT-Nd	BiT-Nb
Crystal system	Orthorhombic	Orthorhombic	Orthorhombic	Orthorhombic	Orthorhombic
Space group	*Aba*2	*Aba*2	*Aba*2	*Aba*2	*Aba*2
T (K)	298	298	298	298	298
*a* (Å)	5.40949(13)	5.40986(23)	5.41282(21)	5.40957(22)	5.41134(15)
*b* (Å)	32.81958(21)	32.81764(16)	32.82626(20)	32.82428(23)	32.82782(31)
*c* (Å)	5.44826(16)	5.44907(30)	5.44637(28)	5.44575(25)	5.44841(16)
*V* (Å^3^)	967.27(4)	967.48(7)	967.72(6)	966.98(6)	967.87(4)
Orthorhombicity	7.15 × 10^−3^	7.22 × 10^−3^	6.17 × 10^−3^	6.66 × 10^−3^	6.83 × 10^−3^
Degree of Texture	89%	91%	89%	82.5%	54%

**Figure 2:**
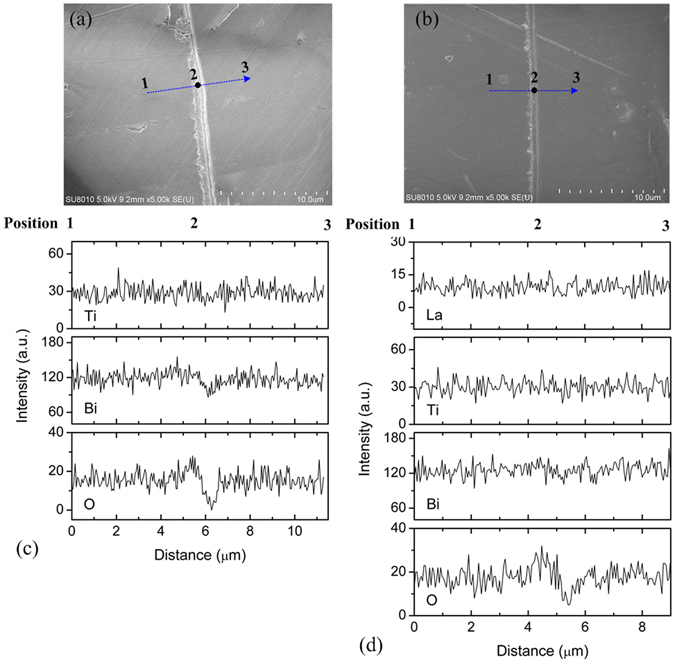
Microstructure of the grain-boundary region of polished (without thermal etching) (**a**) BiT-Bi and (**b**) BiT-La. EDS line scans (line 1 → 3) across the grain-boundary for (**c**) BiT-Bi and (**d**) BiT-La.

**Figure 3:**
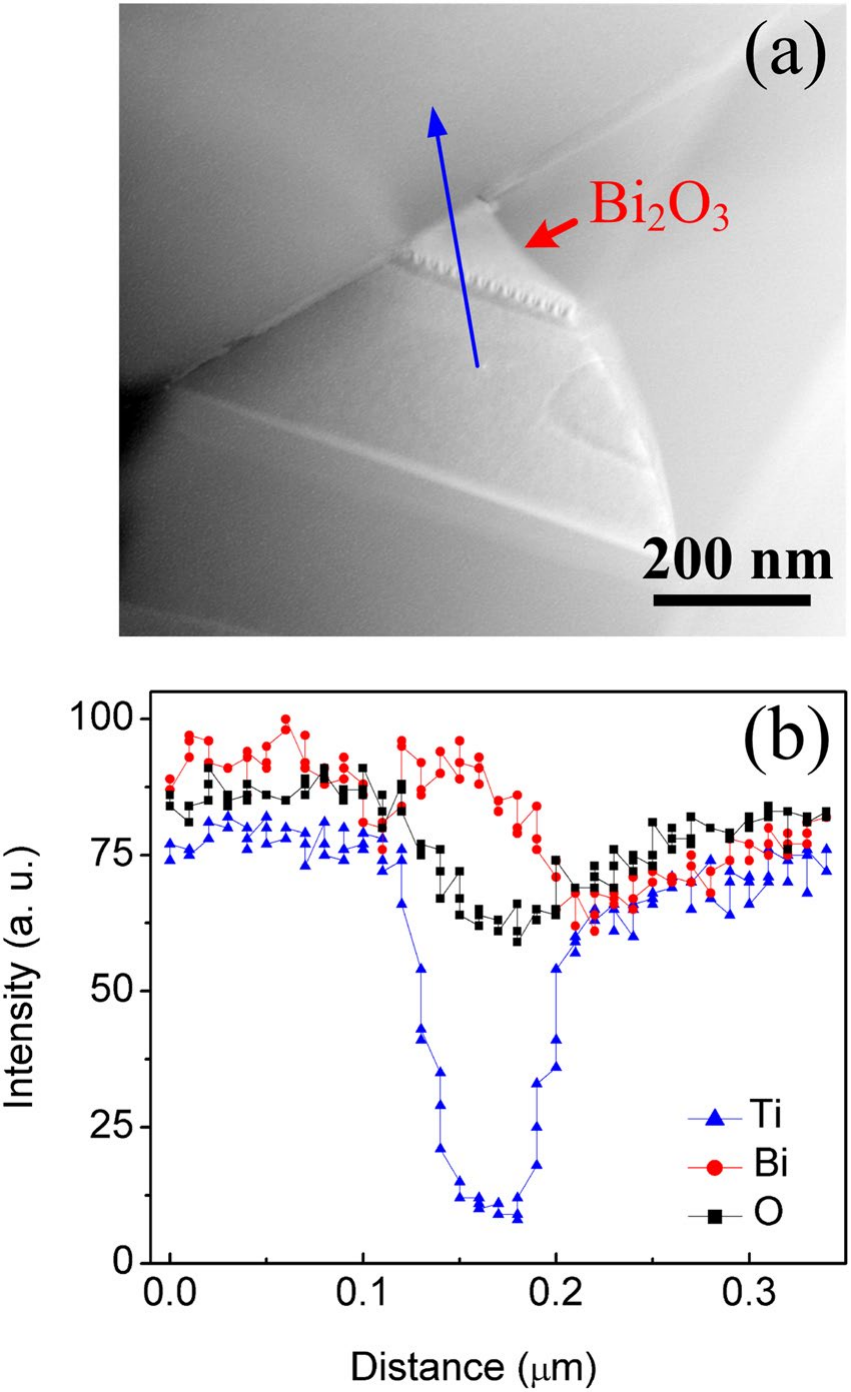
HAADF Z-contrast image showing Bi-rich phase (arrowed light areas) at the triple points in BiT-Bi. (**b**) EDS line scan across the Bi-rich phase in BiT-Bi.

As indicated by surface SEM (scanning electron microscope) images (Fig. S1a–c; Supplementary Information), the great majority of grains in BiT-A are overlarge and laminated. The aspect ratio *L*/*t* (length/thickness) of the laminated grains ranges from 10 to 30. Probably, that stacking and laminating a few such grains leads to high degree of texture in them. In contrast, BiT-Nb shows size-smaller grains, which is consistent with the results of the Ta/Nb substituted BiT ceramics^25^. And no pores are found within grains, which are visible in BiT-A (cross-section SEM images; insets of Fig. S1a–d, Supplementary Information). It is presumed that the incorporation of Nb^5+^ into the lattices leads to a lower grain-growth rate in the *a-b* plane due to low V_O_^••^ concentration ([V_O_^••^]) in the perovskite layers. Figure 2a and b show microstructure of the grain boundary region of the polished BiT-Bi and BiT-La samples, respectively. It is clear that heterogeneous structure is observed at the grain boundaries. As indicated by EDS (energy-dispersive x-ray spectroscopy) line scans, BiT-Bi and BiT-La show no obvious variation in Bi and Ti intensities along the line 1 → 3, whereas a composition deviation with respect to oxygen is observed at the position 2 of grain boundary (Fig. 2c and d). This position may correspond to the grain-boundary surface layer, which is possibly created by the accumulation of space charges. In addition, grain composition for them is analyzed by EDS surface scans of five entire grains, and the average atomic ratios are normalized and listed in Table 2. The measured compositions of BiT-Bi and BiT-La are close to the theoretical ratios in BiT within instrument resolution and standard deviations. However, stoichiometric composition shows somewhat deviation at the position 2 of grain boundary, where the O concentration and the ratio of 2[O]/(3[Bi] + 4[Ti] + 3[La]) are both higher than those in grain (about 63% and 1, respectively). The experiments also reveal about 0.55 at. % La in the bulk phase, close to nominally excess level (0.62 at. %). Presumably, these nonstoichiometric compositions at the grain boundary regions are not associated with Bi_2_O_3_ or La_2_O_3_. TEM (Transmission electron microscopy) image reveals an evidence of some Bi-rich regions at the triple points in BiT-Bi, as shown in Fig. 3a. The relevant EDS line scan reveals the absence of element Ti in Bi-rich phase (Fig. 3b), which is likely ascribed to be Bi_2_O_3_.

**Table 2:** Composition analyses for BiT-Bi and BiT-La. EDS data for grain composition were obtained by surface scans on five entire grains, and the mean value and standard deviation were listed.

Sample	elements	grain	Point 2 at grain boundary	theoretical composition
BiT-Bi (Bi_4_Ti_3_O_12_ • 0.06Bi_2_O_3_)	O (at. %)	63.44(0.38)	66.77	63.11
	Bi (at. %)	21.36(0.41)	20.15	21.35
	Ti (at. %)	15.20(0.46)	13.08	15.54
BiT-La (Bi_4_Ti_3_O_12_ • 0.06La_2_O_3_)	O (at. %)	63.34(0.45)	65.24	63.11
	Bi (at. %)	20.83(0.32)	20.55	20.73
	Ti (at. %)	15.28(0.42)	13.79	15.54
	La (at. %)	00.55(0.21)	00.43	0.62

Point 2 at grain boundary was shown in Fig. 2a and b.

### Impedance spectra and component response

**Figure 4:**
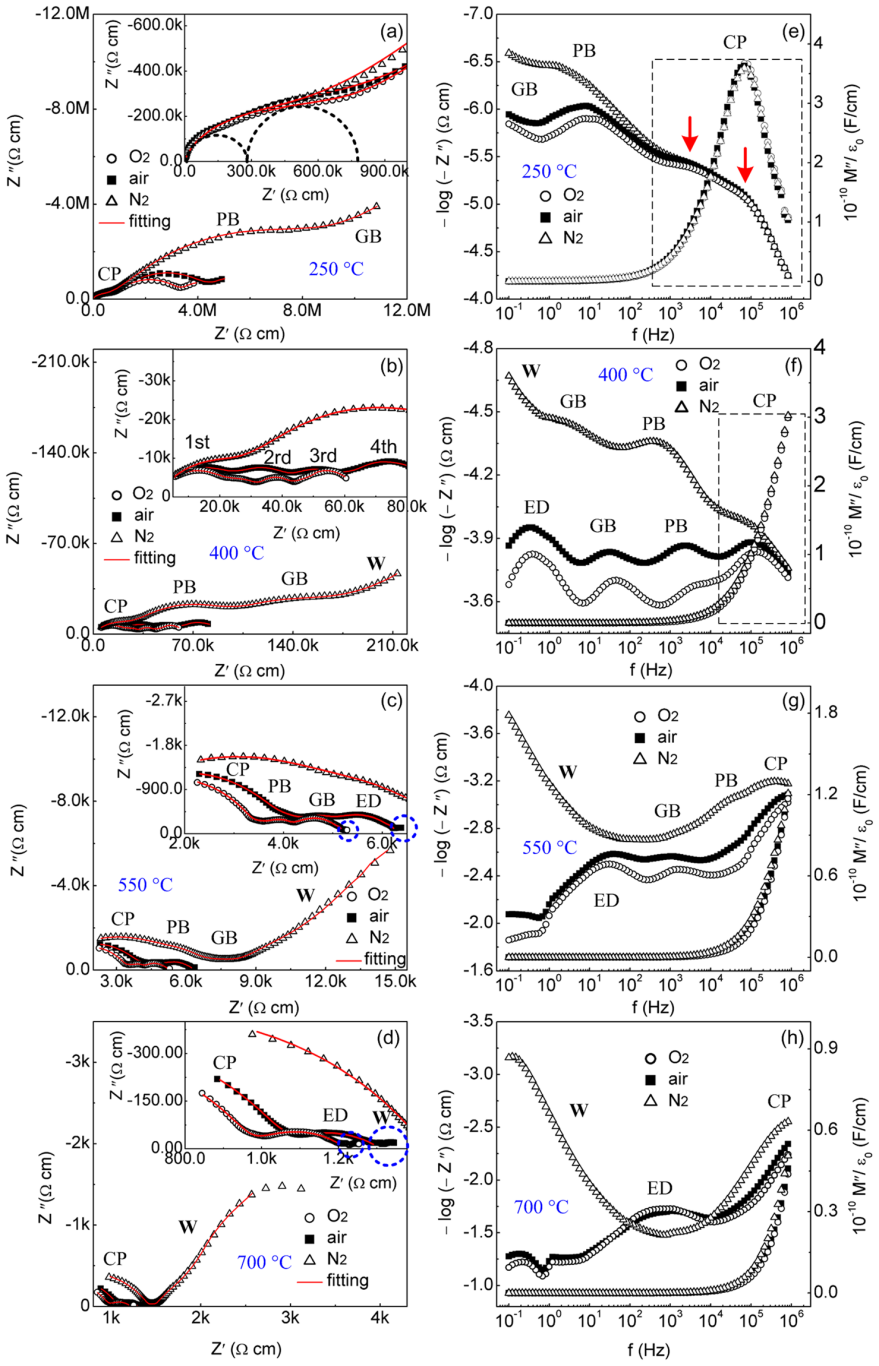
(**a**–**d**) 250–700 °C complex Z* plots of the air-, O_2_- and N_2_-processed BiT-Bi samples, and insets showing regionally enlarged drawings (the symbols: experimental data; the solid lines: fitting spectra). (**e**–**h**) 250–700 °C *Z*″/*M*″ plots of air-, O_2_- and N_2_-processed BiT-Bi samples. “CP” crystalline plate, “PB” plate boundary, “GB” grain boundary, “ED” electrode impedance, “W” Warburg impedance.

**Figure 5:**
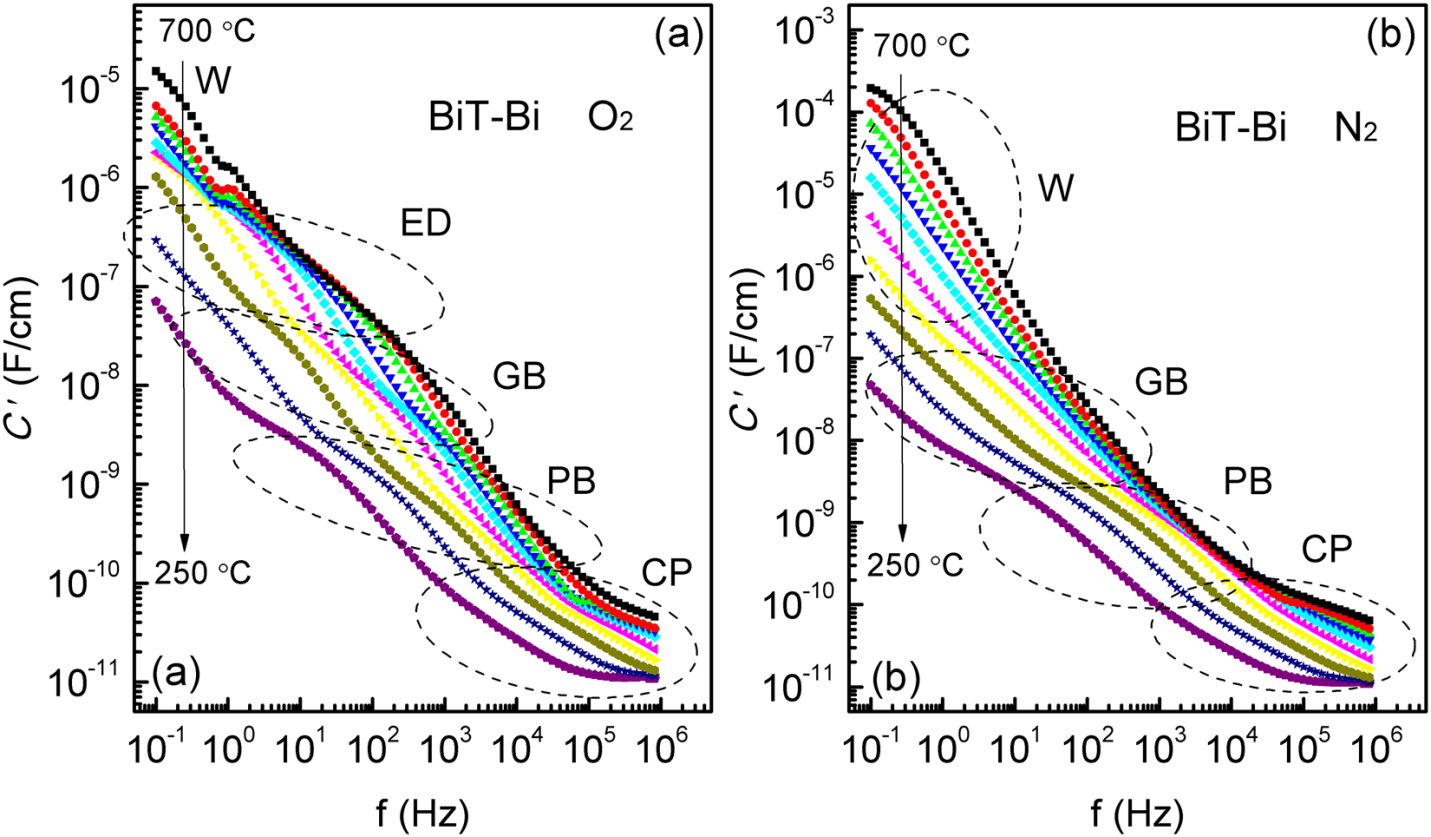
250–700 °C *C*′ spectroscopic plots of the (**a**) O_2_- and (**b**) N_2_-processed BiT-Bi samples.

Figure 4 demonstrates variable-temperature impedance diagrams of the air-, O_2_- and N_2_-processed BiT-Bi samples. For the three samples, two complete arcs at high and medium frequencies and, an incomplete one at low frequencies (0.1–1 Hz) due to the limited measurement range of the instrument, are observed in the 250 °C diagrams (Fig. 4a). The 250 °C capacitance (*C*′) plots of BiT-Bi show a plateau with capacitance values of ∼5 nF/cm at low frequencies (Fig. 5). Therefore, the incomplete arc can be ascribed to a grain boundary (GB) response^48^. Previously, BiT single crystals showed two resolved impedance semicircles, which were considered as the results of the effects of crystalline plate (CP) and plate boundary (PB), respectively^43^. In this study, BiT-Bi ceramic also presents mica-like grains, where CP and PB are visible (inset of Fig. S1a; Supplementary Information). Therefore, the two complete arcs as mentioned above are likely to be associated with the responses of CP and PB, respectively. As indicated by inset of Fig. 4a and the frame inside Fig. 4e, two component parts are observed at the CP region. In BiT-Bi, the electrical properties of the (Bi_2_O_2_)^2+^ layers should be pronounced due to high degree of texture in the *c* axis direction. Therefore, it is likely that they correspond to the AC responses of the (Bi_2_O_2_)^2+^ layers (higher frequency) and the pseudo-perovskite blocks (lower frequency), respectively^49^. It is reasonable that the former has a lower electrical capacitance than the latter, because of the polarization vector of BiT along the crystallographic *a*-axis (within the pseudo-perovskite blocks).

When temperature is elevated, the grain boundary arc becomes complete, and another arc (the 4th arc; inset of Fig. 4b) is visible in the 400 °C air/O_2_ impedance diagrams, at low frequencies (Fig. 4b). The same data presented as Z″ plots (Fig. 4f) also exhibit an additional peak at low frequencies. The 400 °C *C*′ plot of the O_2_ sample shows a high capacitance plateau (10^−7^–10^−6^ F/cm) in the same frequency range (Fig. 5a). These experiments suggest that the most likely origin of the 4th arc is ascribed to the electrode effect (discussed later)^38, 42^. However, a clear Warburg impedance contribution can be observed below 1 Hz in the 400 °C Z^*^ plot of the N_2_ sample (Fig. 4b). To further increasing temperature, such a Warburg response presents an intensive inclined spike at 550 °C and 700 °C (Fig. 4c and d), due to limited ionic diffusion into a partially blocking electrodes^42, 50^. This, combined with steeply increased capacitance at low frequencies (Fig. 5b), indicates that the N_2_ sample shows prominent ionic conduction, and the principal conducting species could be O^2−^ ions^51^. In contrast, the air/O_2_ samples only show a tail associated with a weak trace of Warburg impedance at the lowest frequencies, due to significantly enhanced electronic conduction^53, 54^. The ionic transference number (*t*_ion_) can be evaluated from these Warburg impedances in the case of ion blocking electrode condition, which was described in the literature^52–54^. *t*_ion_ in N_2_ is roughly evaluated to be about 0.85 at 550 °C, which are much higher than those in air and O_2_ (<0.1). High *t*_ion_ in N_2_ is apparently associated with high [V_O_^••^] in the sample.

**Figure 6:**
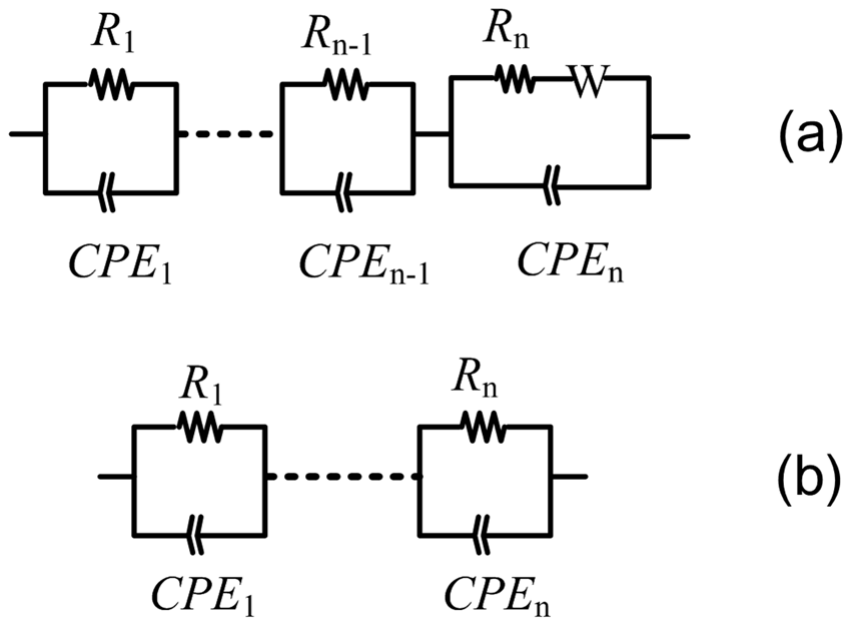
Equivalent circuits to fit the impedance data of BiT and BiT-A under (**a**) N_2_ and (**b**) air/O_2_. “*R*”, “*CPE*” and “*W*” denote resistance, constant-phase element and Warburg impedance, respectively.

In general, the time constant difference for relaxation of each component is large at low temperature, so one can see separated semicircles for each component (see Fig. 4a and b)^55^. However, at high temperature, the time constants for different component are very close and/or relatively small for some of the components, so we normally observe two component semicircles at 700 °C. Like electrolytes and solid-state ionic conducting materials^56, 57^, a modified Randles equivalent circuit was used to calculate the impedance data of the N_2_ sample, as shown in Fig. 6a An equivalent circuit including several (*R//CPE*)s in series (Fig. 6b) was used to calculate the impedance data of the air/O_2_ samples. Here, the bulk is integrated and considered as a (*R//CPE*) component. The parameters and errors for the equivalent circuits are reasonable, and the calculated data well agree with the experiment ones. Similar impedance behaviors as functions of atmosphere and temperature are found for BiT-La and BiT-Nd, which are not shown here. For BiT-La with Pt and Ag electrodes, there are both four overlapping arcs in the 450 °C Z* plots (Fig. S2, Supplementary Information). The resistance (the 4th arc) and the capacitance associated with the electrode effect in the low frequency range are clearly affected by the electrode materials, because of different work functions of them. However, the bulk response that dominates the high frequency data remains unchanged. These experiment results are consistent with those for CaCu_3_Ti_4_O_12_ ceramics^38^. Slightly affected boundary responses may be ascribed to two different preparation conditions for Ag and Pt electrodes.

**Figure 7:**
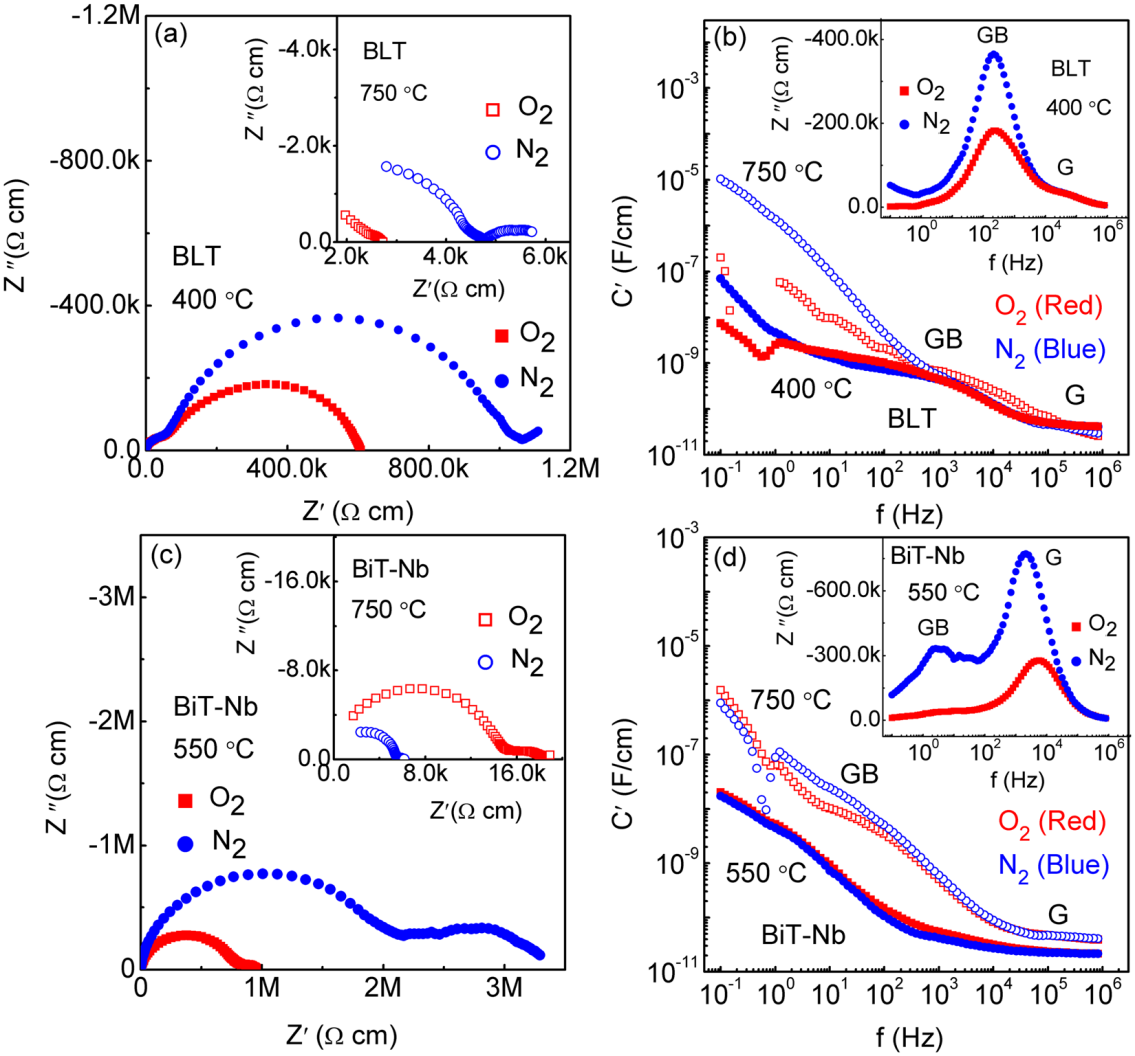
(**a**) 400 °C and 750 °C (inset) Z* plots of the O_2_- and N_2_-processed BLT samples. (**b**) 400 °C and 750 °C *C*′ plots with an inset showing 400 °C *Z*″ plots, of the O_2_- and N_2_-processed BLT samples. (**c**) 550 °C and 750 °C (inset) Z* plots of the O_2_- and N_2_- processed BiT-Nb samples. (**d**) 550 °C and 750 °C *C*′ plots with an inset showing 550 °C *Z*″ plots, of the O_2_- and N_2_-processed BiT-Nb samples.

In contrast, BLT and BiT-Nb show dissimilar AC responses, as shown in Fig. 7. In Fig. 7a, the N_2_-processed BLT sample shows a small inclined spike at 400 °C, which turns into a distorted arc at 750 °C (inset of Fig. 7a). While no obvious Warburg characteristic is found in BiT-Nb at both 550 °C and 750 °C (Fig. 7c and inset of Fig. 7c), which is an indication of a very small contribution of ion conduction to the electrical conduction. This could be related to low [V_O_^••^] and highly stabilized oxygen ion in it. In addition, two principal semicircles are observed in the complex Z^*^ plots of BLT and BiT-Nb under O_2_ and N_2_. The same data presented as *C*′ (Fig. 7b and d) and Z″ (insets of Fig. 7b and d) plots show two capacitance plateaus and two Z″ peaks, respectively. It means that the resistances and the capacitances of BLT and BiT-Nb are principally derived from two regions, *i*.*e*., grain (high frequency) and GB (low frequency), respectively.

### pO_2_ dependences of resistivity(ρ)and conductivity(σ)

**Figure 8:**
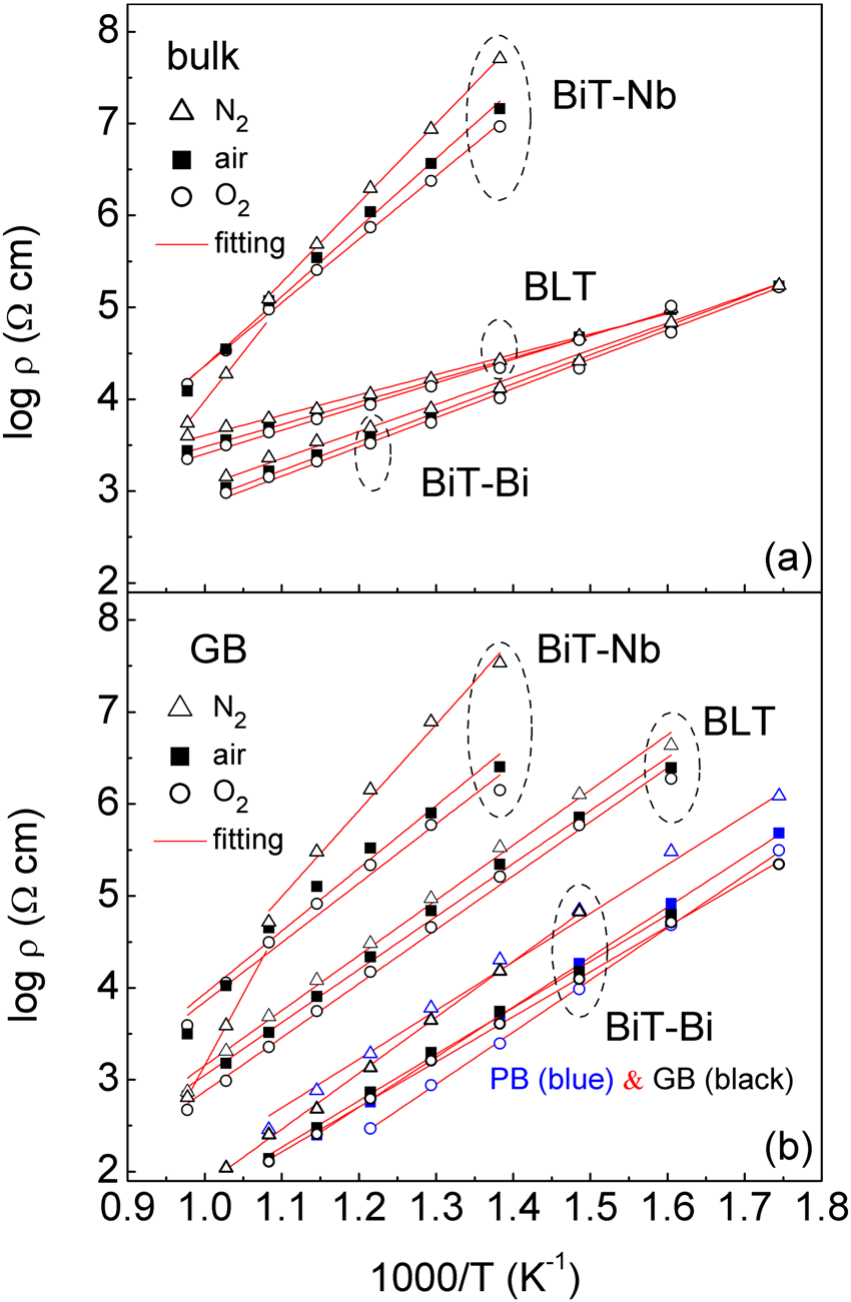
(**a**) ρbu_lk_ and (**b**) *ρ*_GB_ Arrhenius plots for the air-, O_2_- and N_2_-processed BiT-Bi, BLT and BiT-Nb samples.

Figure 8 shows *ρ*_bulk_ (bulk resistivity) and *ρ*_GB_ (GB resistivity) Arrhenius plots for BiT-Bi, BLT and BiT-Nb measured under different atmospheres. As shown in Fig. 8a, BLT shows slightly higher *ρ*_bulk_ than BiT-Bi. While its *ρ*_GB_ increases obviously, by about two orders of magnitude (air-processed) (Fig. 8b). By comparison, BiT-Nb shows excellent electrical insulating property, whose *ρ*_bulk_ and *ρ*_GB_ are both much higher than those of BiT-Bi by approximately three orders of magnitude. In addition, the *ρ*_GB_ of BiT-Bi and BLT is strongly atmosphere-dependent, whereas the *ρ*_bulk_ of them is nearly independent of *p*O_2_. By and large, both *ρ*_bulk_ and *ρ*_GB_ of the samples increase with decreasing *p*O_2_, indicating that *p*-type conduction is predominant in them. However, *n*-type conduction dominates BiT-Nb above *T*_c_, and therefore the *ρ*_bulk_ and *ρ*_GB_ of the N_2_ sample are much lower than those of the air/O_2_ samples above 650 °C. BiT-Bi and BLT show comparable activation energy (*E*_a_) for the bulk and the grain boundary barrier, being 0.5–0.6 eV and 1.0–1.2 eV, respectively. The two values of BiT-Nb become much higher, being 1.37–1.71 and 1.28–1.83 eV, respectively.

**Figure 9:**
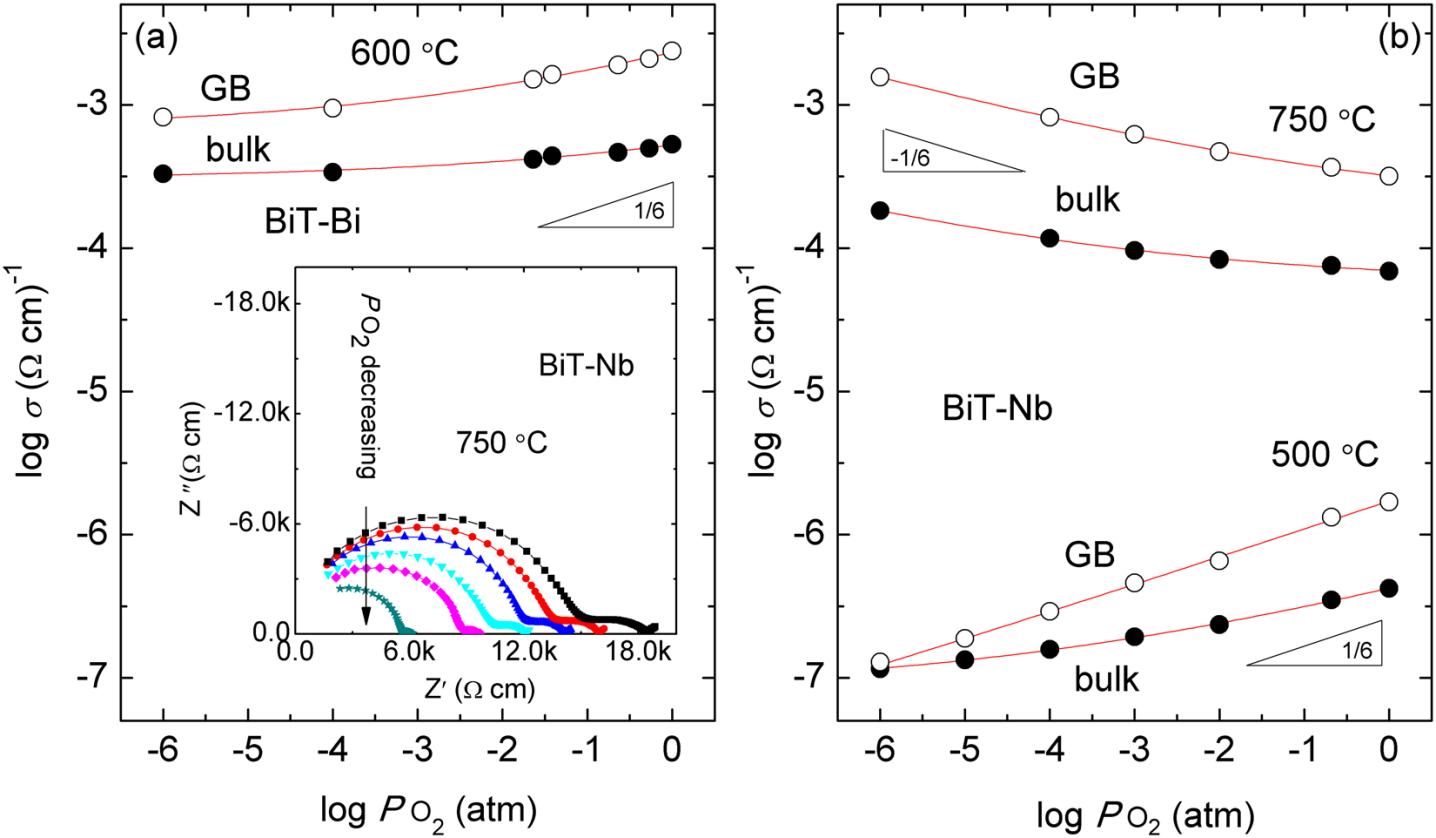
pO_2_ dependences of *σ*_bulk_ and *σ*_GB_ of BiT-Bi and BiT-Nb. (**a**) BiT-Bi at 600 °C, (**b**) BiT-Nb at 500 °C and 750 °C.

Figure 9 shows *σ*_bulk_ (bulk conductivity) and *σ*_GB_ (GB conductivity) as a function of *p*O_2_ for BiT-Bi and BiT-Nb at different temperatures. In Fig. 9a, the *σ*_bulk_ and *σ*_GB_ of BiT-Bi both increase with increasing *p*O_2_, and log*σ*_bulk_ vs log *p*O_2_ and log*σ*_GB_ vs log *p*O_2_ both present nonlinear variations. It is clear that ionic conductivity is approximately independent of *p*O_2_, while hole conductivity linearly increases with increasing *p*O_2_^31, 32^. These nonlinear variations suggest that BiT-Bi is an oxide ionic and *p*-type mixed conductor. By comparison, *σ*_bulk_ showed a very smaller dependence of *p*O_2_, indicating that ion conduction dominates the bulk. As indicated by Fig. 9b, the σ_bulk_ and σ_GB_ of BiT-Nb show strong dependence of oxygen activity, which is in response to a prominent electronic conduction. In the present oxides, oxygen vacancies mainly arise from the evaporation of Bi_2_O_3_ during sintering, and the incorporation of oxygen into the V_O_^••^ sites follows after cooling, expressed by the following reaction.

2 $${V}_{O}^{\bullet \bullet }\frac{1}{2}{O}_{2}\to {O}_{O}^{x}+2{h}^{\bullet }$$

Each V_O_^••^ is compensated by two holes (*h* ^•^) to satisfy the electroneutrality condition, and consequently the sample presents *p*-type conduction. When the reaction by Eq. 2 is dominant, Takahashi, *et al*. suggested that hole conductivity (or hole concentration [*h* ^•^]) follows the 1/6 power dependence of *p*O_2_^31, 32^. The σ_GB_ nearly proportional *p*O_2_^1/6^ in the measured *p*O_2_ range at 500 °C, implies that *p*-type conduction is predominant at the GB of BiT-Nb. However at 750 °C, the two conductivities both increase with decreasing *p*O_2_, which is directly opposite to the experiments exhibited at 500 °C. This change is consistent with the results of YMnO_3_ ceramics^58^. It is presumed that BiT-Nb above *T*_c_ is an oxide ionic and *n*-type mixed conductor. Under reducing conditions the required electrons (*e*′) are created through the release of oxygen from the lattices, expressed by the following equation.

3 $${O}_{O}^{x}\to {V}_{O}^{\bullet \bullet }+2e^{\prime} +\frac{1}{2}{O}_{2}$$

The reduction of Ti^4+^ to Ti^3+^ is likely to be the source of conduction. And the electrons are probably trapped by the donor defects such V_O_^••^ and Nb_Ti_^•^, leading to high *E*_a_ (>2.0 eV) for the N_2_ sample in the high temperature range (see Fig. 8). In addition, good fittings for the *σ*_bulk_ and *σ*_GB_ of BiT-Nb at 750 °C can be obtained by an equation of *σ*_bulk_ (*σ*_GB_) = *σ*_ion_ + *σ*_electronic_ (*p*O_2_)^*m*^ (*m* = −1/6). In the case of both 500 °C (BiT-Nb) and 600 °C (BiT-Bi), the exponent *m* is equal to 1/6. For the O_2-_processed BiT-Bi sample at 600 °C (*p*O_2_ = 1 atm), the calculated *σ*_ion_ and *σ*_electronic_ (hole) in the bulk are 3.02 × 10^−4^ and 2.21 × 10^−4^ S cm^−1^, respectively, and they are estimated to be 0.68 × 10^−3^ and 1.64 × 10^−3^ S cm^−1^ at the GB region, respectively.

### Phase transition and dielectric relaxation

**Figure 10:**
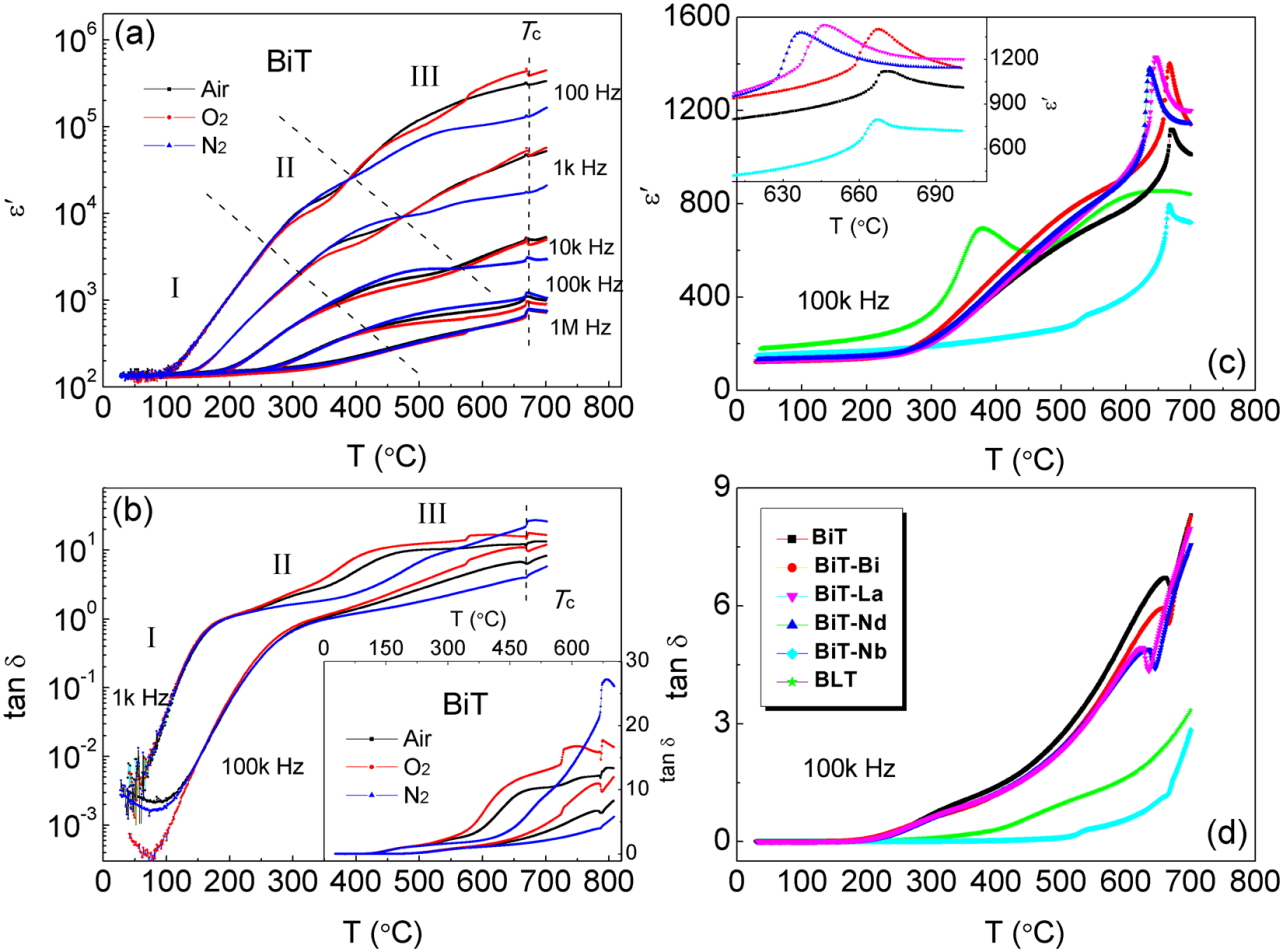
(**a**) Log *ε*′ vs. *T* and (**b**) log tan*δ* vs. *T* plots for the air-, O_2_- and N_2_-processed BiT samples, and tan*δ* vs. *T* plots for the air-, O_2_- and N_2_-processed BiT as an inset in (**b**). (**c**) *ε*′ vs. *T* and (**d**) tan*δ* vs. *T* plots for the air-processed BiT, BiT-A, BLT and BiT-Nb samples, with an inset showing regionally enlarged drawing for *ε*′ vs. *T* plots in (**c**).

Figure 10a shows temperature dependence of dielectric permittivity (*ε*′) at several frequencies for the air-, O_2_- and N_2_-processed BiT samples. High-and-sharp dielectric permittivity peak corresponds to ferroelectric-to-paraelectric phase transition. It is observed that the atmosphere has no influence on *T*_c_ of BiT, which is around 673 °C and the same as those reported in the literature^6, 40^. Notably, an anomaly associated with two primarily high permittivity regions is detected below *T*_c_, which agrees with the results of Shulman *et al*.^12^. Also, there are several peaks observed in the tan *δ*-*T* plots (Fig. 10b), corresponding to the steep increases of dielectric permittivities in the *ε*′ vs. *T* plots (Fig. 10a), which all move toward higher temperature with increasing frequency. The dielectric relaxation and the loss peak in the low temperature range (region I) are not almost affected by altering *p*O_2_, indicating an intrinsically physical nature, *i*.*e*., the bulk response. In contrast, the medium- and high-temperature dielectric relaxations in regions II and III, respectively, are strongly affected. They are likely in response to the boundary capacitance and the electrode capacitance, respectively, being referred to the results of Fig. 4 and ref. 38. The notable peak between 450 and 550 °C in the dielectric loss tangent (inset of Fig. 10b) for the air/O_2_ samples seems to be attributed to the conduction loss.

As shown in Fig. 10c and d, the Nb donor substitution suppresses the loss and the relaxation process dramatically, and a sharp transition peak is observed at *T*_c_. However, the A_2_O_3_ additions are not critical to the dielectric data. In addition, the *T*_c_ of BiT-Bi and BiT-Nb is close to that of BiT, while the *T*_c_ of BiT-La and BiT-Nd decreases to 638 °C and 647 °C, respectively (Fig. 10c). For A-site bismuth-containing Aurivillius compounds, the polarity Bi^3+^ with 6 s^2^ lone pair electrons causes the deformation from the prototype structure^59^. Therefore, it is presumable that non-polarity La^3+^ and Nd^3+^ were incorporated into the lattices by replace the A-site Bi^3+^, and thus the *T*_c_ of BiT-La and BiT-Nd decreases significantly. Newnham *et al*. proposed that *T*_c_ vs. *x* in Bi_4−x_ RE_x_Ti_3_O_12_ follows a linear relationship^60^. In this study, the substitution content *x* in BiT-La and BiT-Nd is roughly estimated to be about 0.1 (Fig. S3; Supplementary Information), close to nominal A-site excess level (Bi_4_A_0.12_Ti_3_O_12.18_). Furthermore, the change trend of *T*_c_ is in accord with that of structural orthorhombicity (see Table 1), and higher *T*_c_ corresponds to higher orthorhombicity value.

## Discussion

**Figure 11:**
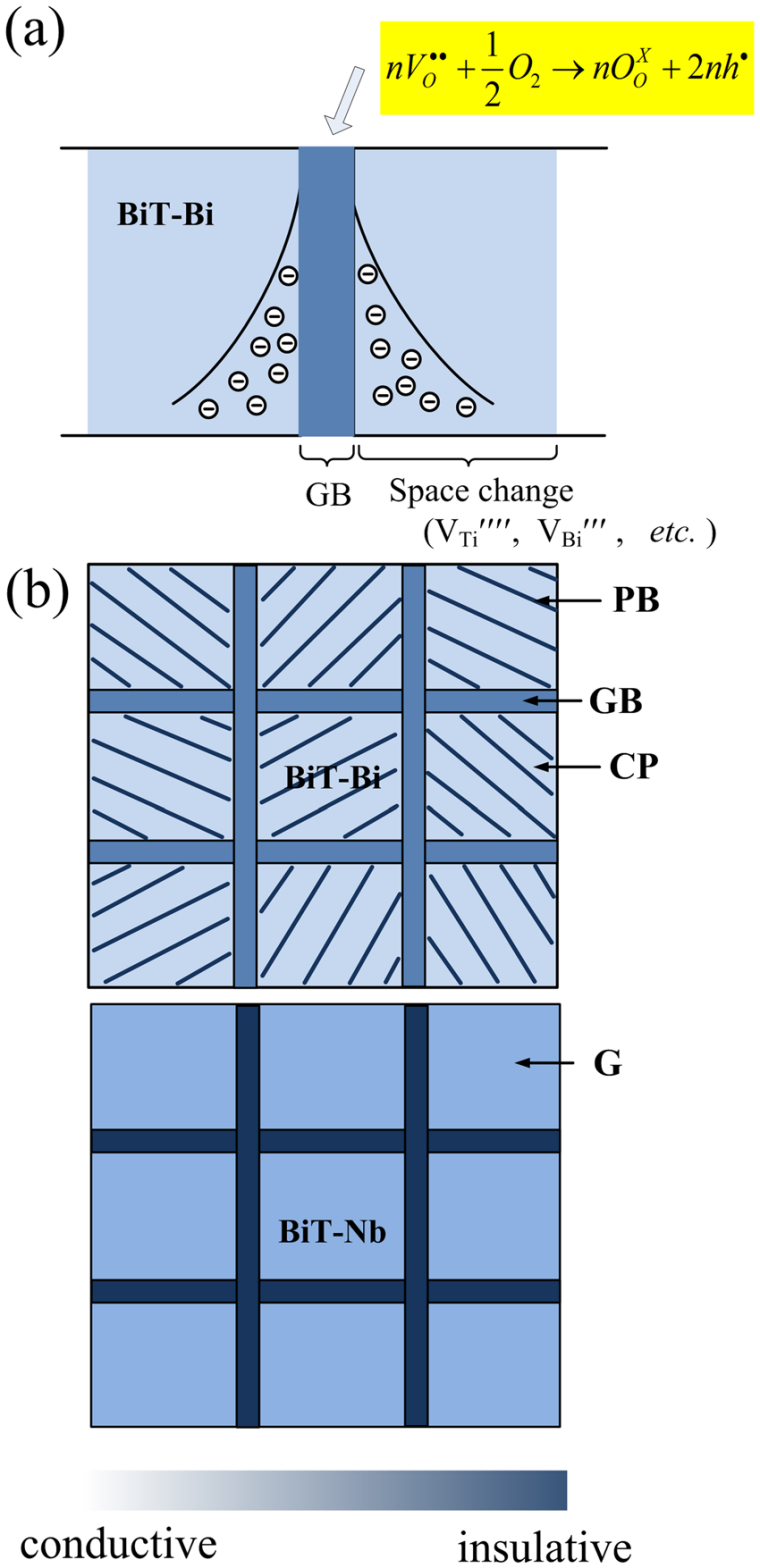
Schematic illustrations of (**a**) formation of the barrier layer at the grain boundary region (**b**) electrical microscopes of BiT-Bi and BiT-Nb.

Commonly, Schottky barriers can form in electrically heterogeneous ceramics at insulating grain boundaries between semiconducting grains, which is referred to as an internal barrier layer capacitor (IBLC) effect^61^. Also, Schottky barriers can occur between “leaky” or semiconducting ceramics and metal electrodes, acting as a non-ohmic electrode effect^62^. The formation of these barriers can be ascribed to compositional variations and/or a mismatch between Fermi energy levels between the two materials that meet at the interface^38, 48^. Polarization effects at these barrier layers can generate nonintrinsic and colossal dielectric permittivities in ceramics such as CaCu_3_Ti_4_O_12_ (CCTO)^38^. In this study, the bulks of BiT-A clearly are semiconducting, whose resistivity are lower than 10^5^ Ω cm above 300 °C (Fig. 8a). Thus the Schottky barriers are possibly generated at the grain boundary regions in them, which is consistent with the results of CCTO and Pb(Fe_1/2_Nb_1/2_)O_3_ ceramics^37, 38^. The results of Figs 8 and 9 indicate that BiT-Bi is an oxygen ion and hole mixed conductor. It is presumed that the bulk of BiT-Bi is dominated by positive defects (*h* ^•^ and V_O_^••^), which can be charge-compensated by grain boundary acceptor surface charges. Such a barrier layer may be associated with the depletion of V_O_^••^ at the grain boundary region during oxidative cooling^63^, as sketched in Fig. 11a.

It is possible that the electrodes nominally “blocking” for ions, allow for a certain ionic leakage, while the electrodes nominally “reversible” for electrons, still represent a certain interfacial resistance^52, 64^. In the case of the air/O_2_ BiT-Bi samples, it is believed that weak Warburg impedance at the lowest frequencies corresponds to the limited ionic diffusion into a partially blocking electrode (see Fig. 4). The additional arc between the boundary impedance and the Warburg one is presumably attributed to the electrode effect due to deviation from electronic reversibility. Two types of electrode effects together with the boundary barrier effects that dominate the intermediate frequency data, induce two high dielectric permittivity regions for BiT-A. Therefore, the following successions of layers starting from the inside are possibly: bulk/plate boundary/grain boundary/electrode.

One can speculate that oxide ion conduction may be a common feature of perovskite materials with a high [V_O_^••^]. Furthermore, high oxygen ion conductivity has been recorded in the literature for intergrowths of Aurivillius with Brownmillerite structure, and cubic δ-Bi_2_O_3_ is known as a fast ion oxygen conductor^11, 65, 66^. For BiT-A, it is reasonable that predominant ionic conduction in the bulk is associated with the textured structure along the *c*-axis direction, in addition to high [V_O_^••^] in the perovskite blocks. However, hole conduction is dominant at the boundaries, and thus the *ρ*_GB_ or σ_GB_ is apparently dependent of *p*O_2_ (see Figs 8b and 9a). A detailed structure analysis for pure BiT showed that some Bi ions in the perovskite layers are overbonded with a valence state of >3+^67^. A Pb^2+^ → Pb^3+^ hopping conduction has been proposed in PZT perovskite^68^. Presumably in BiT-Bi, hole conduction is associated with oxidation of Bi^3+^ to Bi^4+^, and the relevant *E*_a_ is probably close to that for the trapping of holes by Pb^2+^ ions, 0.26–0.3 eV^11, 68, 69^. The reported *E*_a_ for the mobility of V_O_^••^ is about 1.0 eV^70, 71^. The *E*_a_ of about 0.6 eV for the bulk of BiT and BiT-A can be attributed to a compromise of oxide ion conduction and hole conduction. Higher *E*_a_ (1.0–1.2 eV) for the boundary barriers may be attributed to trapping of holes (*h* ^•^) by the grain-boundary acceptor surface charges (A′)^52^.

**Figure 12:**
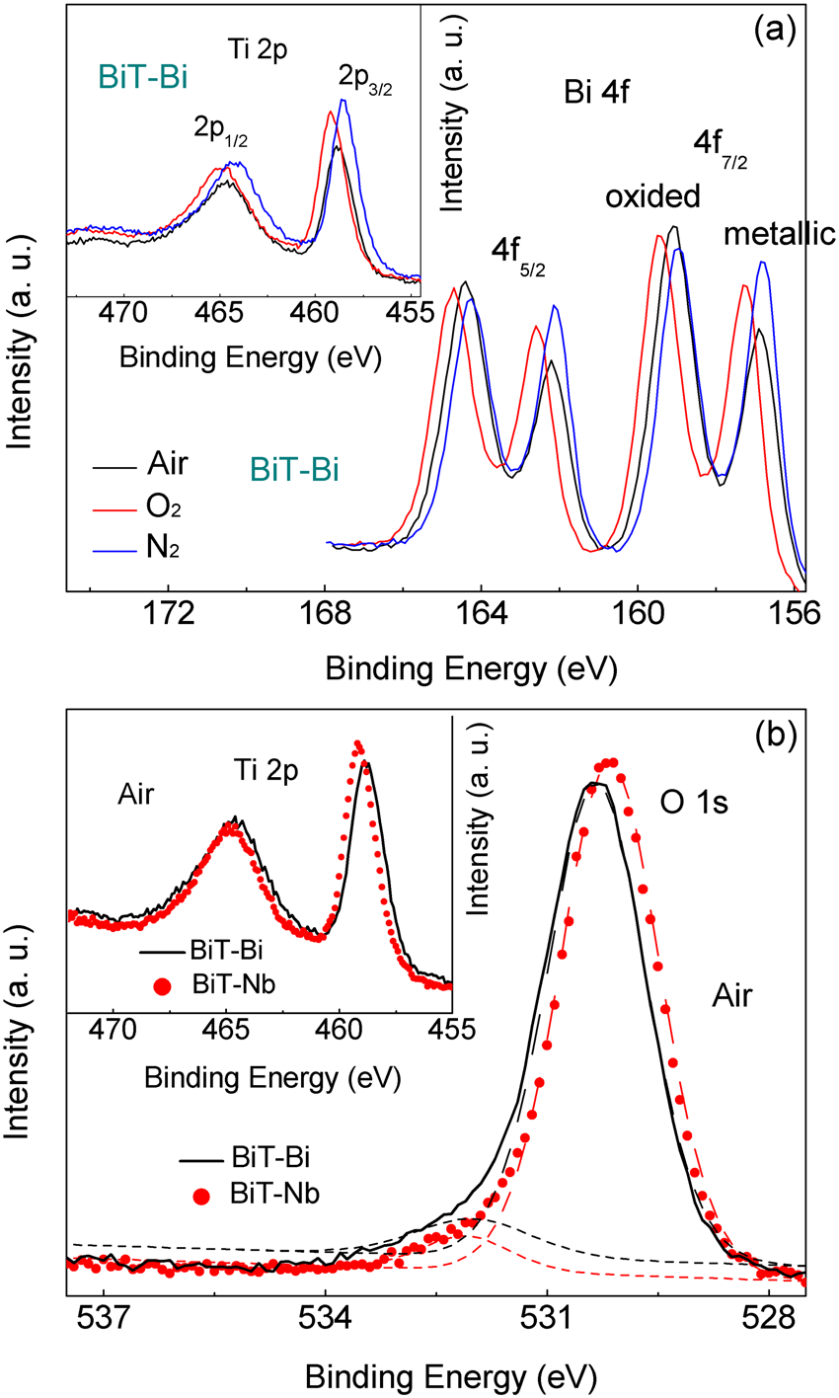
(**a**) Bi *4f* and Ti 2*p* (inset) XPS spectra of the air, O_2_ and N_2_ BiT-Bi samples. (**b**) compared O 1 *s* and Ti 2*p* (inset) XPS spectra of the air BiT and BiT-Nb, where the O 1 *s* spectra were deconvoluted into two natural components by Gauss-Lorentz method (solid lines are experimental data, and dotted lines are fitting peaks).

In order to achieve predominant oxygen ion conduction, it is necessary to avoid samples picking up oxygen from high *p*O_2_ environments. Otherwise, the rich-oxygen atmosphere encourages the incorporation of foreign oxygen into the lattices (Eq. 1); as a consequence, the samples transform into *p*-type electronic conductors with higher conductivity and lower activation energy. As indicated by the XPS (X-ray photoelectron spectroscopy) results (Fig. 12a), the increased *p*O_2_ leads to the increases in the nominal valence states of Bi and Ti, which is related to both a decrease in [V_O_^••^] and an increase in [*h* ^•^]^72^. Therefore, BiT-A show a transition from the dominant oxide ion conduction to the dominant *p*-type semiconduction with increasing *p*O_2_.

As shown in Fig. S4 (Supplementary Information), BiT shows similar conduction behaviors as functions of atmosphere and temperature. BiT-A relative to BiT shows slightly increased *ρ*_bulk_, and the variations in *ρ*_PB_ (PB resistivity) and *ρ*_GB_ are slight and irregular (Fig. S5; Supplementary Information). This, combined with the evolutions in *V* and *T*_c_ (see Table 1 and Fig. 10c), indicates that A^3+^ could be mainly diffused into the lattices by substituting the A-site Bi^3+^, which limited the predicted compensation effect of excess A_2_O_3_ on the V_Bi_′′′ and V_O_^••^ sites. Excess or substituted bismuth (in fact, Bi_2_O_3_), should act as sintering aids because of low melting point (about 830 °C), and consequently BiT-A and BiT show comparable grains and texture factor values. La isovalent substitution with a high concentration can induce an increase in the formation energy of V_O_^••^ in the perovskite blocks, where the decreases in [V_O_^••^] and [*h* ^•^] are induced^33, 73^. Thus in BLT, hole conduction and oxygen ion conduction are both suppressed. As sketched in Fig. 11b, BiT-Nb relative to BiT-Bi becomes rather more insulating. Two situations can be considered by the Nb substitution for the Ti site (B site), as expressed by the following equations.

4 $$N{b}_{2}{O}_{5}+{V}_{O}^{\bullet \bullet }\mathop{\longrightarrow }\limits^{Ti{O}_{2}}2N{b}_{Ti}^{\bullet }+5{O}_{O}^{X}$$

5 $$N{b}_{2}{O}_{5}+2{h}^{\bullet }\mathop{\longrightarrow }\limits^{Ti{O}_{2}}2N{b}_{Ti}^{\bullet }+4{O}_{O}^{X}+0.5{O}_{2}$$

**Figure 13:**
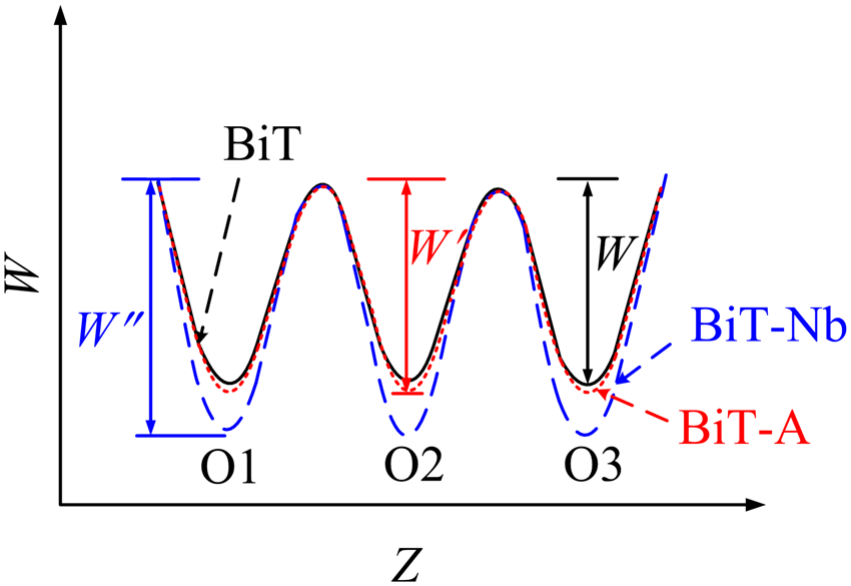
Schematic potential well systems for the jump of oxygen vacancy inside BiT, BiT-A, and BiT-Nb.

Clearly the Nb^5+^ donor substitution can decrease efficiently [*h* ^•^] and [V_O_^••^] in the perovskite blocks, which supports significantly increased *ρ*_bulk_ of BiT-Nb (see Fig. 9). As indicted by the results of Figs 8 and 9, for BiT-Bi (>350 °C) and BiT-Nb, the grain resistivity of is lower than the grain resistivity. However, it does not mean that grain boundaries are electrically more conduction than bulk grains. In fact, grain boundaries in this study are electrically more insulating as shown in Fig. 11b, because the thicknesses of them are far smaller than the grain sizes of samples. This is consistent with the results of other electroceramics^73^. The XPS results of Fig. 12b indicates that BiT-Nb shows lower [V_O_^••^] and stronger Ti-O band than BiT-Bi, in the perovskite blocks. In addition, the donor centrals (Nb^5+^) may affect the movement of V_O_^••^^11, 12^. This combined with low [V_O_^••^], should result in much higher potential barrier energy for the jump of V_O_^••^ in BiT-Nb than those in BiT and BiT-A, as sketched in Fig. 13. Therefore, the oxygen ion conduction is weak in BiT-Nb, of which the electrical conduction is primarily attributed to an electronic mechanism. Previous researches revealed that additional polarization associated with the electrode-sample interface was readily observed for CCTO ceramics with relatively low *ρ*_GB_^38^. If *ρ*_GB_ was large, the electrode polarization was obscured by sample-related effects. Thus, the absence of the electrode effect in BLT and BiT-Nb may be associated with high *ρ*_GB_ of them, which are much higher than those of BiT and BiT-A (see Fig. 8b).

## Conclusions

Structure and electrical properties of modified BiT-based ceramics could be tailored readily by changing chemical compositions, including the perovskite A and B sites. As a consequence, different electrical conductions and dielectric properties among them were found. Excess A_2_O_3_ had no obvious effects on electrical resistivity and conduction mechanism. BiT and BiT-A all showed prominent oxide ion conduction under N_2_, which was likely associated with high [V_O_^••^] in the perovskite blocks and the pronounced texture in the *c*-axis direction. However, the hole conduction was prominent in the air/O_2_ samples, because of the absorption of oxygen by them. In addition to the semiconducting bulk, impedance data revealed that Schottky barriers had been formed both at grain boundary and at the sample-electrode interface in heterogeneous BiT and BiT-A, which contributed to two abnormally high *ε*′ regions below *T*_c_. BLT with 0.75 La substitution at A site and BiT-Nb with 0.03 Nb substitution at B site, both showed a suppression in electrical conduction including ionic and electronic. Especially, a low level of Nb donor substitution led to the significant decreases in [V_O_^••^] and [*h* ^•^]; thus BiT-Nb showed much higher *ρ* and *E*_a_ than BiT and BiT-A. The results in this study are significant for BiT-base high-temperature piezoelectric sensors in solving the origin of high leakage conductivity. Also, they provided some probability for the future work that BiT-based ceramics are considered as new oxide ion conductors by adjusting appropriately chemical compositions.

## Methods

### Materials

BiT-based ceramics, BiT, BiT-A (A = Bi, La and Nd), BLT and BiT-Nb, were prepared by using traditional solid state reaction. Appropriate amounts of starting materials [Bi_2_O_3_ (99.5%), TiO_2_ (99.99%), La_2_O_3_ (99.99%), Nd_2_O_3_ (99.99%), and Nb_2_O_5_ (99.99%); Sinopharm Chemical Reagent Co., Ltd, CN] were mixed and milled in ethanol for 24 h. The mixtures were dried and then calcined at 800 °C for 4 h. The calcined powders were remilled in ethanol for 24 h, dried, ground and cold isostatically pressed into pellets at 300 MPa. The pressed pellets were then sintered at 980–1000 °C for 2 h.

### Characterization and measurements

XRD data were collected by using an automated diffractometer (X’Pert PRO MPD, Philips, Eindhoven, The Netherlands) with a nickel filter (Cu Kα radiation) at room temperature. SEM images were observed by using a field-emission scanning electron microscopy (JEOL-6700F, Japan Electron Co., Tokyo, Japan) instrument equipped with an EDS system, at room temperature. TEM image and compositional analysis were performed by using a transmission electron microscopy (Tecnai F30, FEI, Hillsboro, OR, USA) instrument equipped with a high angle annular dark-field (HAADF) detector and an EDS system, at room temperature. XPS data were performed with a spectrometer (VG ESCALAB220i-XL, Thermo Scientific, Surrey, UK) with Al Kα (*E* = 1486.6 eV) radiation, at room temperature. Temperature dependences of dielectric permittivity and dielectric loss were measured by using an LCR meter (4284 A, Agilent, CA, USA). Impedance data were performed by using an Impedance Analyzer (Solartron, SI 1260, Hampshire, U.K.), with a frequency range of 0.1–1 M Hz and an AC measuring voltage of 0.3 V. The same sample was used for electrical property measurements firstly in air, subsequently in N_2_, and finally in O_2_, at a slow cooling rate. *p*O_2_ dependence of conductivity was measured in the range of 10^−6^–1 atm, which was controlled by mixing O_2_ and N_2_ gases and monitored by a zirconia oxygen sensor. Ag and Pt electrodes for the measurements of the electrical properties were made of fired-on silver paste at 850 °C and sputtered at room temperature (after removing Ag), respectively.

## Electronic supplementary material

Supplementary Information
