# Supplementary material for: Differences in nature of electrical conductions among Bi4Ti3O12-based ferroelectric polycrystalline ceramics
Source: Sci Rep. 2017 Jun 23;7:4193. doi: 10.1038/s41598-017-03266-y (PMC5482841; doi:10.1038/s41598-017-03266-y)
Supplement: Supplementary file 1 — Supplementary Information [file 41598_2017_3266_MOESM1_ESM.pdf]

Supplementary Information for

Differences in nature of electrical conductions among $Bi_4Ti_3O_{12}$-based ferroelectric polycrystalline ceramics

Changbai Long [1, 2], Qi Chang [2] & Huiqing Fan [2]

[1]Science and Technology on Plasma Dynamics Lab, Air Force Engineering University, Xi'an 710038, PR China

[2]State Key Laboratory of Solidification Processing, School of Materials Science and Engineering, Northwestern Polytechnical University, Xi'an 710072, PR China

Corresponding Author:

E-mail: L. C., longchangbai@126.com; F. Q., hqfan3@nwpu.edu.cn.

Tel.: +86 29 88494463; **Fax:** +86 29 88492642.

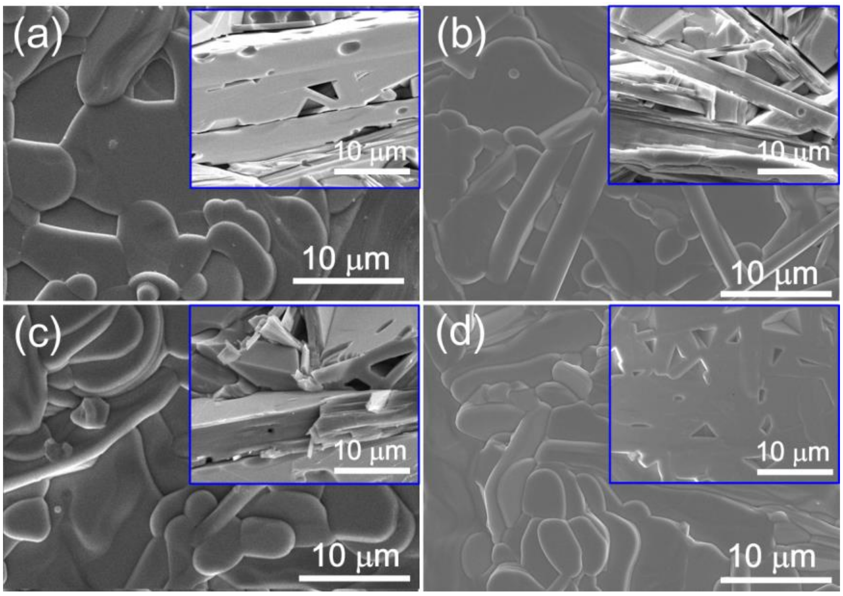

Fig. S1 Surface and cross-section (inset) SEM images of (a) BiT-Bi (b) BiT-La, (c) BiT-Nd, and (d) BiT-Nb ceramics.

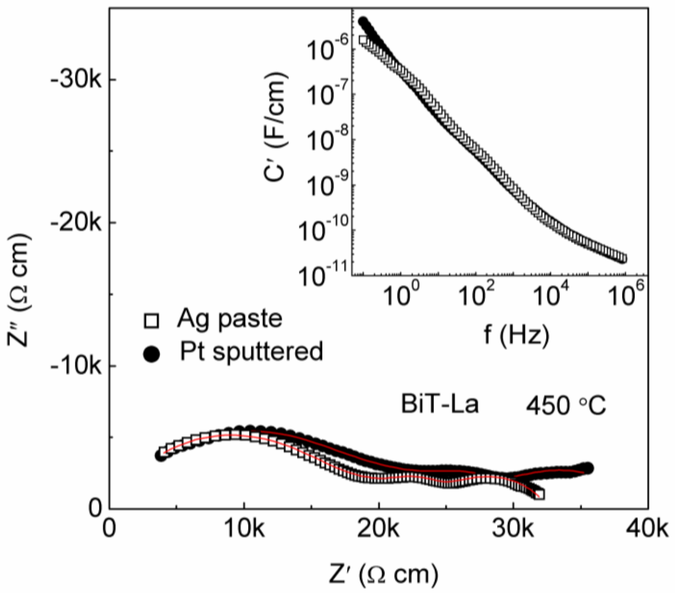

Fig. S2 Complex Z* s and *C*′ spectroscopic (inset) plots at 450 ℃ for the air-processed BiT-La sample with different electrodes (Ag paste and sputtered Pt).

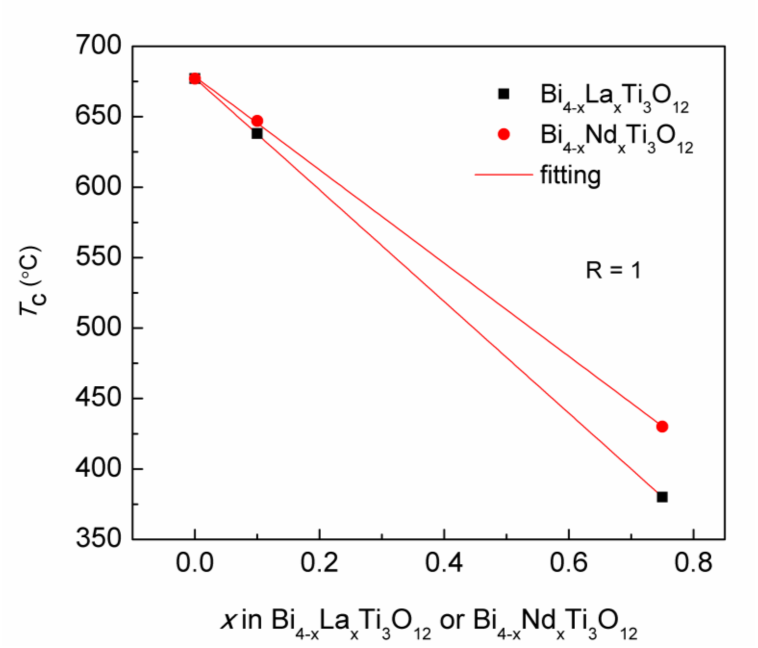

Fig. S3 T_c vs. x in $Bi_{4-x}La_xTi_3O_{12}$ and $Bi_{4-x}Nd_xTi_3O_{12}$

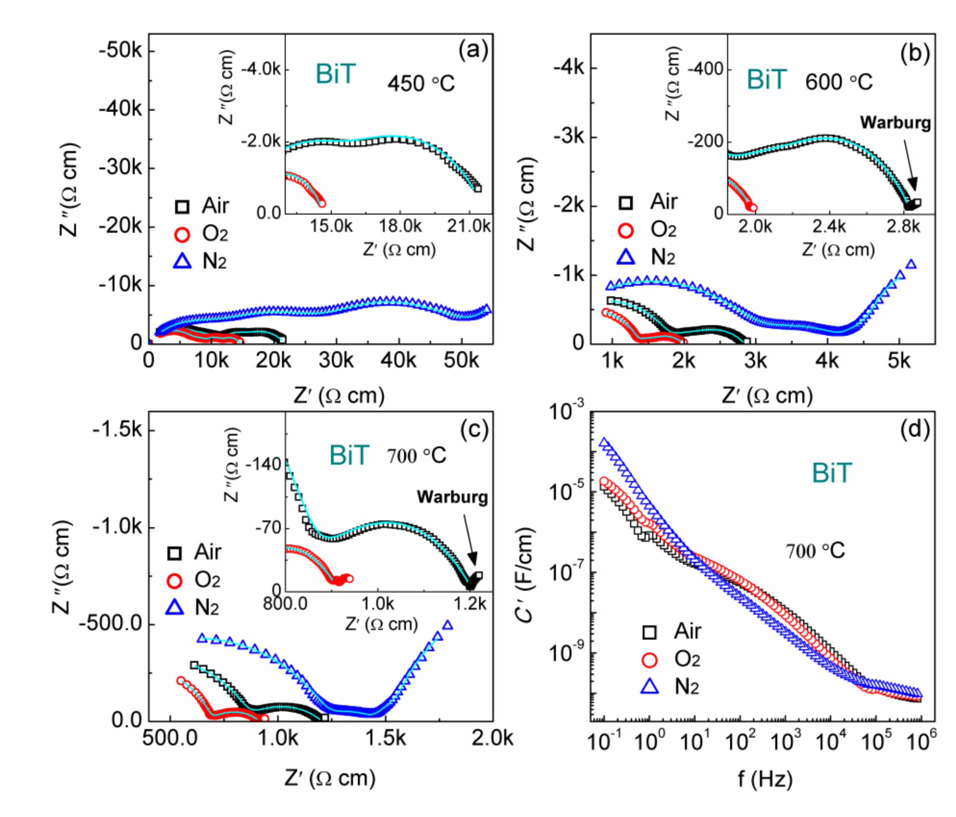

Fig. S4 (a) 450 ℃, (b) 600 ℃ and (c) 700 ℃ *Z** plots of the air-, O_2- and N_2-processed BiT samples, with insets showing the regionally enlarged drawings. (d) 700 °C *C*′ spectroscopic plots of the air-, O_2- and N_2-processed BiT samples.

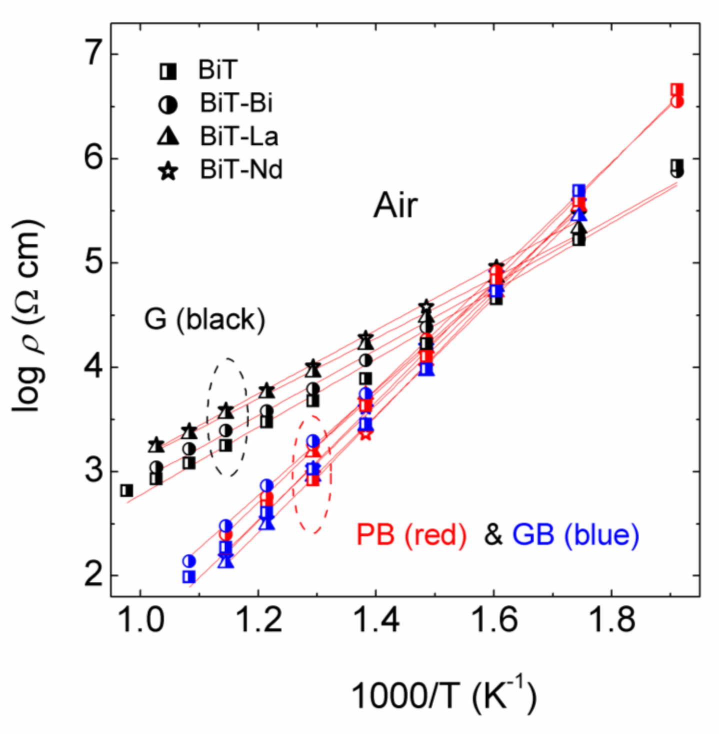

Fig. S5 Bulk, PB and GB resistivity Arrhenius plots for the air- processed BiT and BiT-A samples.
